# TSN-Interworked Deterministic Transmission over WLAN

**DOI:** 10.3390/s25185660

**Published:** 2025-09-11

**Authors:** Woojin Ahn

**Affiliations:** Department of Railroad Electrical and Information Engineering, Korea National University of Transportation, Uiwang 16106, Republic of Korea; woojin.ahn@ut.ac.kr; Tel.: +82-31-460-0575

**Keywords:** wireless LAN, time-sensitive network (TSN), deterministic transmission, scheduled traffic

## Abstract

Many Time-Sensitive Networking (TSN) workloads require deterministic service across heterogeneous links, yet commodity WLANs are contention-based. Although IEEE 802.11be introduced Restricted Target Wake Time (r-TWT) for prioritized access, its ability to robustly guarantee determinism in mixed deployments with legacy devices remains unverified. We propose a standards-aligned scheme that composes r-TWT, Quiet Time Period (QTP), and an optional Randomized Enqueue (RE) policy. These three mechanisms act in concert to protect the Scheduled Traffic (ST) service period (SP) while minimizing the impact on Non-Scheduled Traffic (NST). To analyze how the proposed scheme impacts existing WLANs, we focus the analysis on how the scheme reshapes the contention period (CP)—where opportunistic capacity is realized—by modeling SP/CP timing with renewal theory and embedding it into an EDCA Markov chain. Simulation results confirm that the proposed scheme protects ST determinism: ST throughput remains pinned to the ceiling with zero observed outage and bounded delay across a wide range of station counts. The proposed scheme minimizes NST throughput degradation in the system-peak throughput range (8–12 stations).

## 1. Introduction

In recent years, a broad class of mission-critical and interactive applications has pushed legacy best-effort IoT deployments toward delay-sensitive operation with bounded latency, low jitter, and low loss. Representative domains include industrial automation, intelligent transportation, smart cities, and media/entertainment, where end devices must exchange data predictably across heterogeneous stacks and networks. This shift is reflected in ongoing standards work on deterministic networking and time-sensitive communication [1,2,3,4].

Ethernet is an attractive substrate for such applications due to its ubiquity and cost efficiency, yet classical Ethernet remains best-effort and thus prone to queuing delays and timing uncertainty. The IEEE 802.1 Time-Sensitive Networking (TSN) suite addresses this gap by adding time synchronization (IEEE 802.1AS), scheduled access via the Time-Aware Shaper (TAS, IEEE 802.1Qbv), and frame preemption (IEEE 802.1Qbu/IEEE 802.3br), enabling deterministic coexistence of scheduled and best-effort traffic on the same physical network [5,6].

Even though TSN is gaining significant attention, it lacks native support for wireless networks, which present unique challenges such as asymmetric channels, unreliable latencies, and interference [7]. In heterogeneous applications that rely on wireless connectivity, such as mobile robotics or connected vehicles, the flexibility and mobility offered by wireless are even more critical than in traditional, static industrial environments. Current research has begun to concentrate on realizing TSN features in the wireless domain, but a mainstream wireless technology for TSN has not yet emerged [8].

Among the candidates for wireless TSN, both 5G and IEEE 802.11 wireless local area network (WLAN) are prominent. 5G networks aim to support TSN through URLLC (Ultra-Reliable Low-Latency Communication) services, focusing on centralized control and network slicing [9]. In contrast, Wi-Fi is considered a strong candidate for its deployment flexibility and cost-effectiveness. However, for WLAN, the primary obstacle is the contention-based channel access mechanism, EDCA (Enhanced Distributed Channel Access). The backoff procedure, fundamental to EDCA, introduces unpredictable jitter in transmission delay, which contradicts the deterministic latency that TSN seeks to achieve [10].

While past amendments of the IEEE 802.11 standard mostly focused on enhancing peak throughput, considerable effort is being made to enhance other aspects such as latency and reliability in the latest amendments. IEEE 802.11ax (Wi-Fi 6) introduced Target Wake Time (TWT), representing a fundamental shift toward scheduled channel access by allowing stations (STAs) to negotiate wake times with the access point (AP). This feature reduces contention and improves energy efficiency, and its potential for dynamic, traffic-aware scheduling has been actively explored [11,12,13]. Building upon this, IEEE 802.11be (Wi-Fi 7) introduced Restricted TWT (r-TWT), which provides exclusive, contention-free channel access during negotiated service periods, making it a key technology for real-time applications (RTAs) [8,14]. Recent studies have proposed mathematical models to optimize these R-TWT parameters to satisfy strict quality of service (QoS) requirements for a dedicated flow [15]. Furthermore, research on low-overhead time synchronization demonstrates ongoing efforts to adapt core TSN components for the wireless medium [16].

While IEEE 802.11ax/11be provides the building blocks for scheduled access (TWT) and exclusive service periods (r-TWT), the literature either studies r-TWT in isolation or does not quantify how to jointly guarantee deterministic Scheduled Traffic (ST) service while bounding the capacity impact on legacy BE traffic. For instance, the analytical work by [15] focuses on a dedicated R-TWT flow in isolation. To our knowledge, no prior work has proposed or analyzed a scheme that synergistically combines r-TWT with the Quiet Time Period (QTP) to realize a wireless equivalent of TSN’s Time-Aware Shaper.

This paper proposes a novel transmission scheme to support time-sensitive traffic in WLAN networks by minimizing transmission jitter. (i) We propose a practical r-TWT + QTP scheduling scheme that functionally emulates TAS—guarding ST slots and suppressing post-SP collision bursts—under standard WLAN. (ii) We develop a closed-form analytical model combining renewal theory for the CP residual life and a Markov chain EDCA analysis for NST. (iii) We also provide a standards-aligned control-plane mapping to TSN centralized user control and centralized network control (CUC/CNC) functional entities for deployability.

The remainder of this paper is organized as follows. In Section 2, we describe the proposed scheme in detail. In Section 3, we present a mathematical model to analyze system throughput. In Section 4, we validate the analysis through simulation. In Section 5, we discuss implementation considerations, limitations, and cross-technology comparisons. Finally, Section 6 concludes the paper.

## 2. The Proposed Scheme

### 2.1. Background: The Time-Aware Shaper (TAS) in TSN

**Figure 1:**
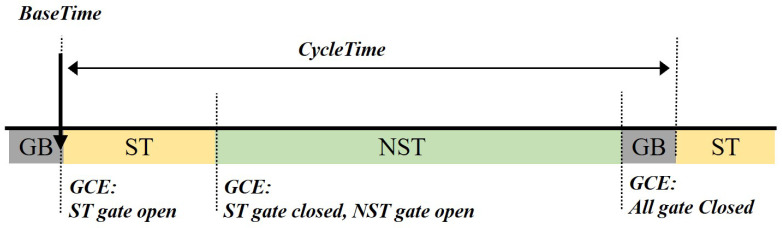
The operational cycle of the TSN Time-Aware Shaper (TAS), illustrating the scheduled slots for ST and NST traffic protected by a guard band.

Among the key features of Time-Sensitive Networking (TSN), the Time-Aware Shaper (TAS), defined in the IEEE 802.1Qbv standard, is a central mechanism that provides deterministic communication capabilities to standard Ethernet [17]. TAS ensures that high-priority Scheduled Traffic (ST) can be transmitted at a predictable time, free from interference by other traffic. As illustrated in Figure 1, its operation is defined by a sophisticated schedule controlled by the following key parameters:

1. Schedule Anchor and Synchronization (BaseTime): All TAS scheduling operates on an absolute time base that is synchronized across the entire network. The BaseTime parameter specifies the absolute, nanosecond-precision time at which the schedule begins its first cycle. All switches in the network, synchronized via the IEEE 802.1AS (generalized precision time protocol (gPTP)) protocol, start their Gate Control List (GCL) operations at this identical BaseTime [18]. This is a core concept that directly corresponds to the Target Wake Time parameter in our proposed wireless scheme, which likewise defines the absolute start time of each service period (SP).
2. Periodic Scheduling (CycleTime): The schedule initiated at BaseTime repeats indefinitely based on a fixed period known as the CycleTime. This periodic nature allows for the continuous and stable handling of ST streams that require data transmission at regular intervals.
3. Gate Control (GateControlEntry): Each cycle is divided into multiple ‘time slots’, and the behavior of each slot is determined by a gate control entry (GCE). A GCE specifies two key pieces of information:
  - TimeInterval: Defines the duration of the time slot.
  - GateState: An 8-bit mask that defines the ‘Open’ or ‘Closed’ state of the gates for each of the eight traffic queues.
  To support ST traffic, the GateState for a specific TimeInterval is configured to open the gate only for the ST-designated queue, while closing the gates for all other lower-priority queues. This creates an exclusive transmission opportunity, where contention is completely eliminated.
4. Guard Band for Interference Prevention: A critical challenge arises when a long, low-priority frame, which started transmission in the preceding time slot, overruns into the ST slot, thereby delaying the time-sensitive frame. To solve this, TAS inserts a guard band before the ST slot. The guard band is a special time slot where the GateState is set to close all gates, ensuring the medium is idle and available right before the ST frame is scheduled for transmission.

By combining a synchronized BaseTime, a periodic CycleTime, precise gate control entries, and protective guard bands, TAS strictly governs the transmission timing of ST frames, enabling deterministic communication in wired Ethernet environments.

### 2.2. Limitations of Existing WLAN Mechanisms

To implement the deterministic communication functions of wired Time-Sensitive Networking (TSN) in an IEEE 802.11 wireless LAN environment, three key technical challenges must be addressed:

1. Traffic Isolation: How can Scheduled Traffic (ST) be isolated from Non-Scheduled Traffic (NST) during medium access contention?
2. Guaranteeing Timeliness: How can a designated ST frame be guaranteed to transmit at its precise scheduled time, without delays caused by other traffic?
3. Network Efficiency: How can the negative performance impact of resource reservation for ST traffic on the overall network be minimized?

This section first examines the limitations of existing WLAN mechanisms in addressing these challenges. Subsequently, it proposes a novel wireless TSN scheme that synergistically combines Restricted TWT (r-TWT) from IEEE 802.11be and Quiet Time Period (QTP) from 802.11ax to overcome these limitations.

#### 2.2.1. The Non-Deterministic Nature of Standard EDCA

**Figure 2:**
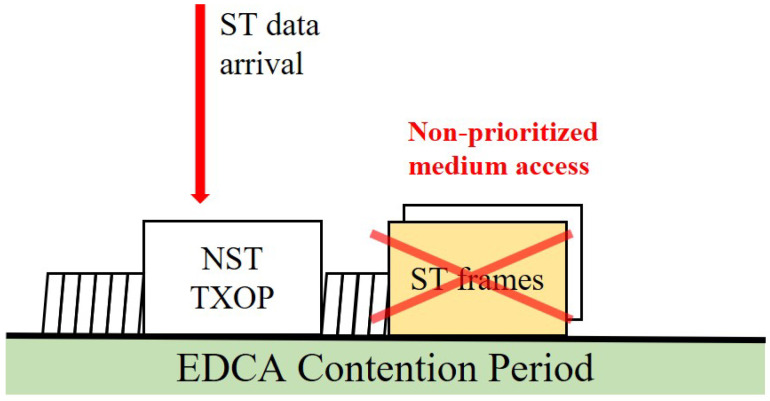
The problem of non-prioritized medium access for ST frames in a standard Enhanced Distributed Channel Access (EDCA) contention period.

The fundamental medium access method in WLAN, Enhanced Distributed Channel Access (EDCA), is inherently contention-based. As depicted in Figure 2, there are no reserved transmission opportunities for ST frames in a standard EDCA environment. An ST frame must contend for channel access through the backoff procedure just like any other NST frame, making it fundamentally impossible to guarantee deterministic, low-latency transmission [19].

#### 2.2.2. Limitations of IEEE 802.11ax TWT

**Figure 3:**
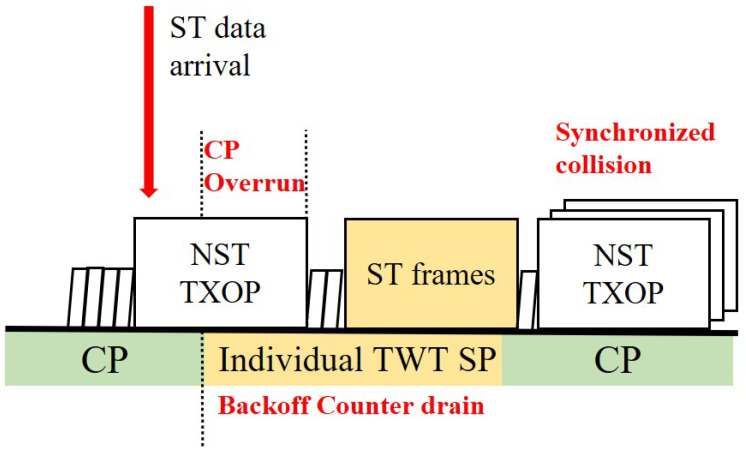
Limitations of legacy TWT: CP overrun and synchronized collision due to backoff counter drain.

The Target Wake Time (TWT) feature introduced in IEEE 802.11ax enables scheduling by assigning stations to specific service periods (SPs). As shown in Figure 3, dedicating an individual TWT for ST frames can achieve isolation from NST traffic, but it introduces two critical problems:

- Contention Period (CP) Overrun: A station that starts a NST frame transmission in the contention period (CP) just before the TWT SP can extend its transmission opportunity (TXOP) into the TWT SP. This ‘CP Overrun’ delays the start of the ST transmission, causing significant jitter that undermines timeliness.
- Backoff Counter Drain and Synchronized Collision: During the TWT SP, other stations are forbidden from transmitting, but their backoff counters continue to decrement whenever the channel is sensed idle. This ‘Backoff Counter drain’ phenomenon causes the backoff counters of multiple stations to converge to zero simultaneously, leading to a massive ‘Synchronized collision’ at the exact moment the next CP begins.

### 2.3. A Novel Transmission Scheme for Deterministic WLAN

**Figure 4:**
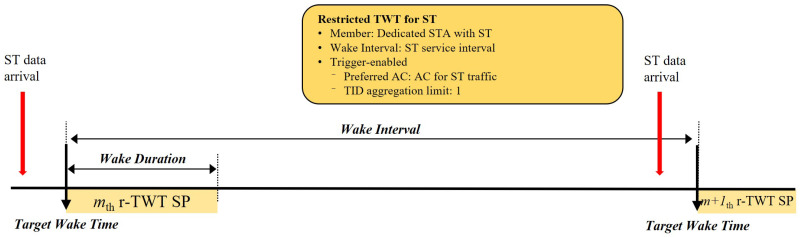
Scheduling architecture of r-TWT.

To overcome the limitations of existing mechanisms, we propose a scheme that orchestrates three features with distinct roles: (1) r-TWT acts as the functional guard band, (2) QTP breaks synchronized collisions inherent to distributed channel access, and (3) Randomized Enqueue smooths the CP’s initial access intensity. The overall scheduling architecture is shown in Figure 4.

#### 2.3.1. r-TWT as a Functional Guard Band (GB)

**Figure 5:**
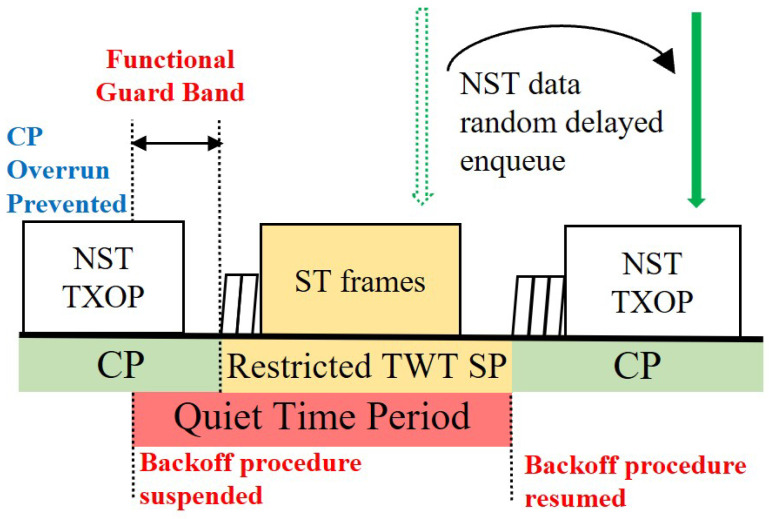
Role separation: r-TWT implements a functional guard band (no CP overrun), QTP suspends backoff for non-participating STAs to prevent synchronized collisions, and Randomized Enqueue spreads buffered NST arrivals to avoid a CP load spike.

Restricted TWT (r-TWT) is an IEEE 802.11be feature for deterministic low-latency service. Unlike standard TWT, r-TWT hard-protects SP boundaries: non-participating STAs are prohibited from starting any TXOP that could overrun into the r-TWT SP. This is functionally equivalent to the guard band in wired TSN, which blocks lower-priority traffic to protect an ST slot [17,18,20]. Hence, r-TWT provides a built-in GB without extra configuration, ensuring the medium is idle at each SP boundary and that the scheduled ST frame can start on time (Figure 5). The r-TWT SP is configured as trigger-enabled so that the AP explicitly solicits the intended ST frame with the dedicated access class or traffic identifier (AC/TID) at SP start, resolving any internal AC contention within the STA [20].

As illustrated in Figure 4, service periods (SPs) are anchored at absolute times and repeat with the ST service interval. Each SP reserves a short window dedicated to the stringent ST flow. We assume (i) a single STA is the r-TWT member for that flow; (ii) the SP is trigger-enabled so the AP explicitly solicits the intended AC/TID at the start; (iii) r-TWT protects the CP→SP boundary (functional guard band), so no CP TXOP can spill into the SP; (iv) early termination: the SP may end immediately after the first successful ACK.

The r-TWT parameters determine the balance between ST protection and NST opportunity. Increasing the SP duration ($T_{\mathrm{sp}}$) strengthens ST protection but shortens the contention period (CP) for NST, while increasing the guard band reduces boundary collisions at the cost of CP airtime. Once an ST transmission is triggered within the SP, it is allowed to complete, but if $T_{\mathrm{sp}}$ is set too short under harsh channels, a failed first attempt may leave insufficient time for retries. In managed industrial or enterprise deployments, administrative controls (for example, bounding TXOP or PPDU sizes near SP boundaries) help maintain guard band efficiency. A quantitative sizing rule for $T_{\mathrm{sp}}$ that meets a target outage while accounting for collisions, packet-error retransmissions, and protocol overhead is developed in Section 3.4; practical adaptive operation, where the AP updates TWT parameters over beacon intervals based on observed packet error rate and ST burst size, is also discussed there.

#### 2.3.2. QTP for Breaking Synchronized Collisions

While r-TWT prevents CP overrun, synchronized backoff remains a problem in distributed access. To desynchronize contenders, we enable the Quiet Time Period (QTP) for the entire r-TWT SP. QTP suspends backoff for non-participating STAs, freezing their counters at current random values [21]. This breaks post-SP synchronized collisions and shortens the idle-to-first-NST gap when CP resumes. In this case, AP is not subject to backoff-suspend—its ACs keep counting down during QTP and, at SP start, the AP transmits a trigger on the minimum-counter AC, yielding near-instant access. Even with r-TWT boundary protection, legacy contenders below 11be (or OBSS neighbors that do not honor r-TWT) can still initiate a TXOP near the SP boundary. Since QTP is defined in 802.11ax and widely implemented, scheduling a short QTP window that begins before the SP also suppresses these legacy attempts, reducing the chance of boundary infringement. This benefit is deployment-dependent; when all contenders are 11be-aware, the incremental gain is small, but in mixed environments it provides additional robustness. In this work we size the effective guard band by the longest NST transmission time on the link, including headers, inter-frame spaces, and ACK. The idea is simple: the guard band must be long enough that even the longest permitted NST TXOP cannot spill into the r-TWT service period. We then align the QTP to begin one such NST transmission time before each service period and to remain active throughout the service period. For clarity in the rest of the paper, we assume all NST ACs share the same airtime; if they differ in practice, the guard band and the QTP lead time should be dimensioned against the largest NST airtime, with a small implementation margin if desired.

#### 2.3.3. Randomized Enqueue for Smoothing CP Load Spikes

Even with desynchronization, NST frames that arrive during the r-TWT SP are buffered and would otherwise be burst-released at CP start, creating a sharp spike in access intensity. We therefore apply a Randomized Enqueue (RE) policy: buffered NST frames are re-enqueued over the subsequent CP at random offsets, spreading their channel access attempts over time (Figure 5). This converts a burst into a smoother process, reducing transient collisions and queue build-up at CP onset. For analytical tractability (Section 3.3.3), RE is reflected by an effective arrival rate in CP, which preserves the total arrivals per service interval while attenuating the instantaneous burst at CP start.

With these three pillars—r-TWT as GB, QTP for desync, and RE for CP smoothing—the scheme delivers deterministic ST service over standard WLAN while bounding the transient and steady-state impact on NST traffic.

## 3. Numerical Analysis

**Figure 6:**
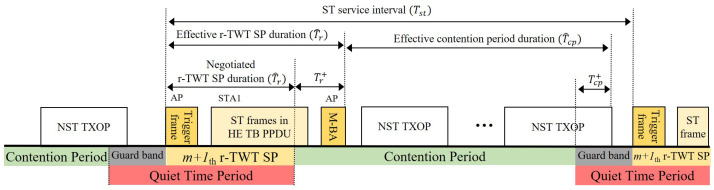
The periodic structure of the proposed scheme for the analytical model.

In this section, we mathematically analyze the performance of the proposed scheme. The analytical model follows the scheme’s periodic operation (Figure 6): Each service cycle comprises a contention period (CP) for NST and a Restricted TWT service period (SP) for ST. We normalize time by the application service interval; hence, one service cycle has duration $T_{st}$, i.e., $T_{\mathrm{cycle}}\equiv T_{st}$. Henceforth we drop $T_{\mathrm{cycle}}$ and use $T_{st}$ only. The functional guard band is provided by r-TWT boundary protection, and a QTP is aligned to start one maximum NST airtime before each SP and remains active throughout the SP. Our analysis derives the expected ST and NST throughputs by modeling the dynamics within these periods.

### 3.1. Analytical Model Overview

The application fixes the service interval, and each r-TWT service period (SP) is anchored at $t_{0}+n\;T_{st}$. We therefore use the service interval itself as the normalization time. Let $B_{st}$ and $B_{nst}$ denote the expected payload bits successfully delivered per service interval by ST and NST traffic, respectively. The long-term system throughput is

(1) $$H=\frac{B_{st}+B_{nst}}{T_{st}}.$$

To obtain $B_{st}$ and $B_{nst}$, we (i) use an outage model for the contention-free ST traffic under r-TWT with early termination, and (ii) adapt a well-established EDCA Markov chain model for NST traffic to the r-TWT/QTP context [22].

### 3.2. Scheduled Traffic (ST) Throughput Analysis

ST data is transmitted during the r-TWT SP without contention, so only channel errors affect success. Because the AP lets every AC keep counting down during the guard band, at least one AC reaches a near-zero counter when the SP starts. The AP immediately sends the trigger frame using that lowest-counter AC, and—since the scheduled ST-STA is the only awake device—faces no external contention. This makes the channel access delay just a few micro-seconds (≪TXOP duration), and we safely ignore it in the ST-throughput model.

Let $p_{e}$ be the packet error rate (PER) per attempt. We distinguish the durations of a successful vs. failed ST attempt inside the SP: $T_{\mathrm{succ},st}$ and $T_{\mathrm{fail},st}$. If the first success occurs at the *k*-th attempt, the time consumed is $S_{k}=\left(k-1\right)T_{\mathrm{fail},st}+T_{\mathrm{succ},st}$. Define

(2) $$K_{\max}≜\max\left\{k\geq 1:\;S_{k}\leq T_{r}\right\},\;\mathrm{with}\;K_{\max}=0\;\mathrm{if}\;T_{r}<T_{\mathrm{succ},st}.$$

An outage occurs if no success finishes within the SP, hence

(3) $$p_{o}=p_{e}^{\;K_{\max}}.$$

Let $L_{st}$ be the ST payload per interval; then the expected ST payload and throughput are

(4) $$B_{st}=\left(1-p_{o}\right)L_{st},\;H_{st}=\frac{B_{st}}{T_{st}}=\frac{\left(1-p_{e}^{\;K_{\max}}\right)\;L_{st}}{T_{st}}.$$

If an SP carries $N_{f}>1$ ST frames (or multiple stringent ST flows), (3) and (4) can be extended by modeling the number of successful frames as a binomial random variable, or by summing independent single-frame terms when independence holds. In this work we focus on the single stringent ST case for clarity.

### 3.3. Non-Scheduled Traffic (NST) Throughput Analysis

NST traffic receives service only during the contention period (CP); hence, its capacity hinges on (i) the effective CP duration per service interval, ${\hat{T}}_{cp}$, and (ii) the contention dynamics inside that CP segment.

#### 3.3.1. Modeling the Effective Contention Period Duration ($E[{\hat{T}}_{cp}]$)

The effective CP duration, ${\hat{T}}_{cp}$, is a random variable that depends on the actual termination time of the preceding r-TWT service period (SP) and on the residual virtual-slot time at the CP boundary. The CP is treated as a renewal process of i.i.d. virtual slots (idle, success, collision), so the boundary overrun $T_{cp}^{+}$ is the residual life of a renewal interval (see (10)). Some implementations may instead release the SP early upon the first ACK (early termination); in that case, an underrun term $T_{r}^{-}$ appears. Consequently, the average effective CP duration is

(5) $$E\left[{\hat{T}}_{cp}\right]\;=\;T_{cp}\;+\;E\left[T_{cp}^{+}\right]\;-\;E\left[T_{r}^{+}\right]\;+\;E\left[T_{r}^{-}\right],$$

where $T_{cp}$ and $T_{st}$ are the negotiated CP length and the negotiated SP length.

(i) r-TWT SP Overrun and Underrun $(E\left[T_{r}^{+}\right]$ and $E[T_{r}^{-}])$

The r-TWT SP gates the start of transmissions: non-participating STAs must finish before the SP, whereas the participating STA, once it starts within the SP, may finish after the SP boundary. Hence underrun exists only if an explicit early termination policy is enabled; otherwise, we set $E[T_{r}^{-}]=0$.

Let $p_{e}$ be the PER per attempt. We distinguish the durations of a successful vs. a failed ST attempt within the SP: $T_{\mathrm{succ},st}$ and $T_{\mathrm{fail},st}$. If the first success occurs at the *k*-th attempt, the time consumed is

(6) $$S_{k}\;=\;\left(k-1\right)\;T_{\mathrm{fail},st}\;+\;T_{\mathrm{succ},st},\;k=1,2,\ldots$$

Define the largest index whose success would finish within the SP as

(7) $$K_{\max}\;≜\;\max\left\{\;k\geq 1:\;S_{k}\leq T_{r}\;\right\},\;\mathrm{with}\;K_{\max}=0\;\mathrm{if}\;T_{r}<T_{\mathrm{succ},st}.$$

(Equivalently, overrun begins at the $\left(K_{\max}+1\right)$-st attempt.)

r-TWT SP overrun occurs when no success finishes within the SP (i.e., $k>K_{\max}$); attempts then continue across the boundary until one succeeds. The expected overrun is

(8) $$E\left[T_{r}^{+}\right]\;=\;p_{e}^{\;K_{\max}}[\underset{\mathrm{residual}\;\mathrm{of}\;\mathrm{the}\;\mathrm{boundary-spanning}\;\mathrm{attempt}}{\underset{︸}{K_{\max}T_{\mathrm{fail},st}+T_{\mathrm{succ},st}-T_{r}}}\;+\;\underset{\mathrm{extra}\;\mathrm{failures}\;\mathrm{entirely}\;\mathrm{after}\;\mathrm{the}\;\mathrm{SP}}{\underset{︸}{\frac{p_{e}}{1-p_{e}}\;T_{\mathrm{fail},st}}}].$$

If $T_{r}<T_{\mathrm{succ},st}$ then $K_{\max}=0$ and $E\left[T_{r}^{+}\right]=\left(T_{\mathrm{succ},st}-T_{r}\right)+\frac{p_{e}}{1-p_{e}}\;T_{\mathrm{fail},st}$.

Underrun is only present if early termination is enabled; otherwise $E[T_{r}^{-}]=0$. If early termination is enabled and success occurs at $k\;\leq \;K_{\max}$, the returned time is $T_{r}-S_{k}$. Taking expectation,

(9) $$E\left[T_{r}^{-}\right]\;=\;\sum_{k=1}^{K_{\max}}\left(T_{r}-S_{k}\right)\;\left(1-p_{e}\right)\;p_{e}^{\;k-1}.$$

(ii) Contention Period Overrun $\left(E\left[T_{cp}^{+}\right]\right)$

The CP is a renewal process formed by i.i.d. virtual slots (idle, success, fail). Since the CP timer can expire at an arbitrary instant inside a slot, the overrun from the CP boundary is the residual life of a renewal interval. This directly yields the inspection paradox result $E\left[T_{cp}^{+}\right]=E\left[T_{\mathrm{vslot}}^{2}\right]/\left(2E\left[T_{\mathrm{vslot}}\right]\right)$, avoiding per-boundary case analysis. We use this as a first-order approximation; the brief non-stationarity right after QTP release is negligible for sufficiently long CPs. Let $T_{\sigma }$ and $T_{\mathrm{vslot}}$ denote the duration of a slot time (Table 1) and a virtual slot (idle, successful TXOP, or collision), respectively. By renewal theory (inspection paradox), the mean boundary overrun equals the mean residual life:

(10) $$E\left[T_{cp}^{+}\right]\;=\;\frac{E\;\left[T_{\mathrm{vslot}}^{2}\right]}{2\;E\;\left[T_{\mathrm{vslot}}\right]},$$

with moments

(11) $$\begin{array}{cc} E[T_{\mathrm{vslot}}] & =\left(1-p_{b}\right)\;T_{\sigma }\;+\;p_{s}\;T_{\mathrm{succ},\mathrm{nst}}\;+\;p_{f}\;T_{\mathrm{fail},\mathrm{nst}},\\ E[T_{\mathrm{vslot}}^{2}] & =\left(1-p_{b}\right)\;T_{\sigma }^{2}\;+\;p_{s}\;T_{\mathrm{succ},\mathrm{nst}}^{2}\;+\;p_{f}\;T_{\mathrm{fail},\mathrm{nst}}^{2}. \end{array}$$

Here $p_{b},p_{s},p_{f}$ are steady-state EDCA slot probabilities (busy, success, fail), and $T_{\mathrm{succ},\mathrm{nst}}/T_{\mathrm{fail},\mathrm{nst}}$ denote the durations of successful/failed NST virtual slots assumed AC-invariant in this work.

#### 3.3.2. Markov Chain Analysis for NST Throughput

**Figure 7:**
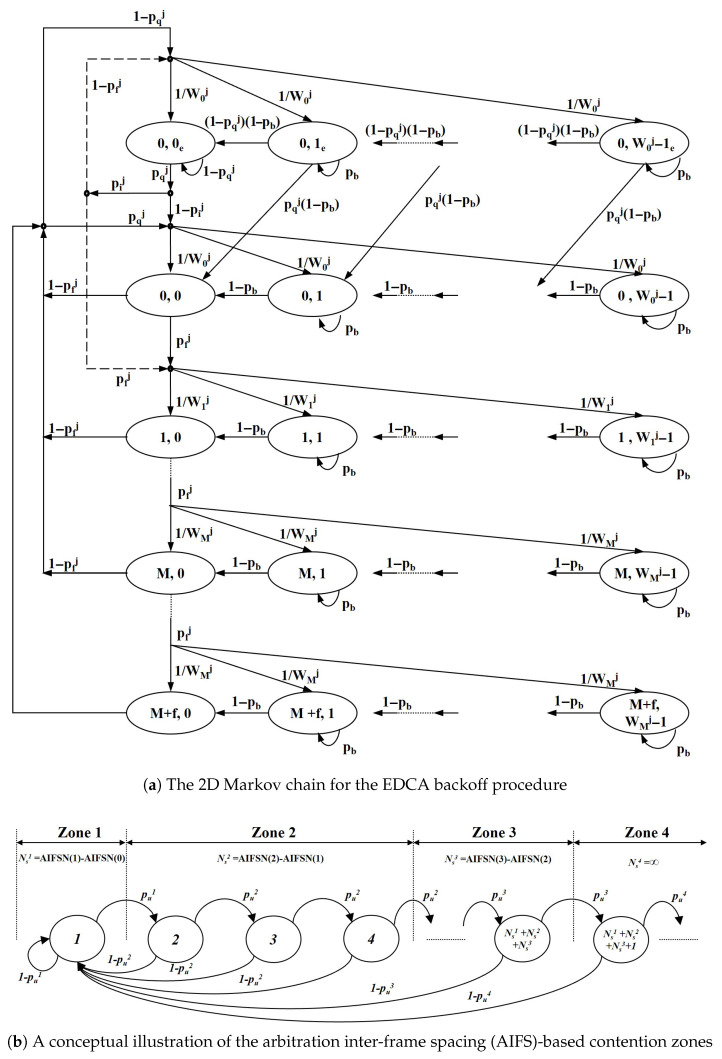
The analytical model for the EDCA mechanism, based on [22].

The throughput of a single NST station, $h_{nst}$, is derived using a per-AC 2D Markov chain model for the EDCA backoff procedure, based on the analytical framework presented in [22]. The model, illustrated in Figure 7, captures the state transitions of a station’s backoff counter. In this section, the AC index *j* ranges over the NST access categories (AC) active in the CP. We use $p_{f}$ for the slot-level fail probability in the vslot moments, whereas $p_{f}^{j}$ denotes the attempt-level failure probability for AC *j* in the Markov fixed point (“collision or channel error”), cf. (12). These are distinct quantities.

The core of the analysis is to solve a non-linear system of equations relating a station’s transmission probability, $\tau^{j}$, to the transmission failure probability, $p_{f}^{j}$, that it experiences. This system is solved using a fixed-point iteration method.

We model per-AC failure as the complement of “collision-free and error-free” success:

(12) $$p_{f}^{j}\;=\;1-\left(1-p_{\mathrm{coll}}^{j}\right)\left(1-p_{e}\right),$$

where $p_{\mathrm{coll}}^{j}$ is the probability that an AC-*j* attempt collides. Equation (12) feeds the fixed-point relation for $\tau_{j}$ in (13).

The stationary distribution of the Markov chain provides a relationship between these key probabilities. For brevity, we present the final key equation here and refer the reader to [22] for the detailed step-by-step derivation. Given the failure probability $p_{f}^{j}$, the transmission probability $\tau^{j}$ is

(13) $$\tau^{j}=\frac{2(1-2p_{f}^{j})}{\left(1-2p_{f}^{j}\right)\left(W+1\right)+p_{f}^{j}W\left(1-{\left(2p_{f}^{j}\right)}^{m}\right)}$$

where *W* is the minimum contention window ($CW_{min}$) and *m* is the maximum backoff stage.

Once the system of equations is solved, the resulting probabilities are used to calculate the average throughput for a single AC:

(14) $$h_{\mathrm{nst}}^{j}\;=\;\frac{p_{s}^{\;j}\;L_{\mathrm{nst}}}{E[T_{\mathrm{vslot}}]}\;,$$

where $L_{nst}$ is the NST payload size and $p_{s}^{j}$ is the success probability for that specific AC.

A key contribution of our work is modeling the impact of QTP on traffic arrival. Traffic that accumulates during the QTP is conserved and spread over the subsequent CP; we therefore normalize by the effective CP length $E[{\hat{T}}_{cp}]$ to obtain $\hat{\lambda }$. This abstraction is independent of whether Randomized Enqueue is enabled. We model the accumulation of NST packets during the quiet period by defining an effective packet arrival rate, $\hat{\lambda_{j}}$:

(15) $${\hat{\lambda }}_{j}\;=\;\lambda_{j}\left(1+\frac{T_{\mathrm{QTP}}}{E[{\hat{T}}_{cp}]}\right)$$

where $\lambda_{j}$ is the original arrival rate. This effective rate is then used to calculate the non-empty queue probability $p_{q}^{j}$ within the Markov model.

(16) $$p_{q}^{j}=1-e^{-\hat{\lambda_{j}}E\left[T_{vslot}\right]}$$

This links our system’s unique dynamics to the established EDCA analysis, allowing for a comprehensive performance evaluation.

#### 3.3.3. NST Throughput Aggregation

Let $N_{j}$ be the number of NST STAs mapped to AC j∈{0,1,2,3} and $h_{nst}^{j}$ the per-AC NST rate (bit/s) obtained from the EDCA Markov chain. Given the effective CP share $E[{\hat{T}}_{cp}]$, the expected NST payload bits per service interval are

(17) $$B_{nst}\;=\;E\left[{\hat{T}}_{cp}\right]\;\sum_{j=0}^{3}N_{j}\;h_{nst}^{j}.$$

Normalizing by the (fixed) service interval $T_{st}$ yields

(18) $$H_{nst}\;=\;\frac{B_{nst}}{T_{st}}\;=\;\frac{E[{\hat{T}}_{cp}]}{T_{st}}\;\sum_{j=0}^{3}N_{j}\;h_{nst}^{j}.$$

#### 3.3.4. Calculation of Key System Probabilities

The slot-level failure probability, $p_{f}$, is derived from two fundamental system probabilities that must be calculated first: the channel busy probability, $p_{b}$, and the aggregate success probability, $p_{s}$. The methodology for calculating these is based on the main reference [22], but is adapted to account for the error-prone wireless channel in our model.

The channel busy probability, $p_{b}$, represents the probability that at least one station is transmitting in an arbitrary time slot. The formula for $p_{b}$ is adopted directly from the reference model. It is derived from the 1D Markov chain for the contention period and depends on the transmission probability $\tau^{j}$ of each station:

(19) $$p_{b}\;=\;\sum_{n}p_{z}^{n}\left[\;1-\prod_{j\in J(n)}{\left(1-\tau_{j}\right)}^{N_{n}^{j}}\right],$$

where $p_{z}^{n}$ is the stationary probability of being in contention zone *n*, and $N_{n}^{j}$ is the number of stations of class *j* in that zone.

The aggregate success probability, $p_{s}$, is the probability that exactly one station transmits successfully in an arbitrary time slot. While the fundamental structure for its calculation is based on the reference model, a crucial modification is required for our wireless environment.

The reference model assumes a perfect channel where a transmission failure is solely due to a collision. Our model, however, considers an error-prone channel. Therefore, a transmission is only truly successful if (1) exactly one station transmits (no collision), and (2) no packet error occurs. This is modeled by multiplying the reference model’s success probability by the probability of no channel error, $\left(1-p_{e}\right)$.

(20) $$p_{s}\;=\;\left(1-p_{e}\right)\sum_{n}p_{z}^{n}\sum_{j\in J(n)}N_{n}^{j}\;\tau_{j}{\left(1-\tau_{j}\right)}^{N_{n}^{j}-1}\prod_{\begin{array}{c} h\in J(n)\\ h\neq j \end{array}}{\left(1-\tau_{h}\right)}^{N_{n}^{h}},$$

The term inside the parentheses is derived from the reference model (e.g., Equation (36) in [22]), and the channel error probability $p_{e}$ is integrated for the analysis of this paper.

Finally, the slot-level failure probability equals the busy probability minus the success probability:

(21) $$p_{f}\;=\;p_{b}-p_{s}.$$

**Figure 8:**
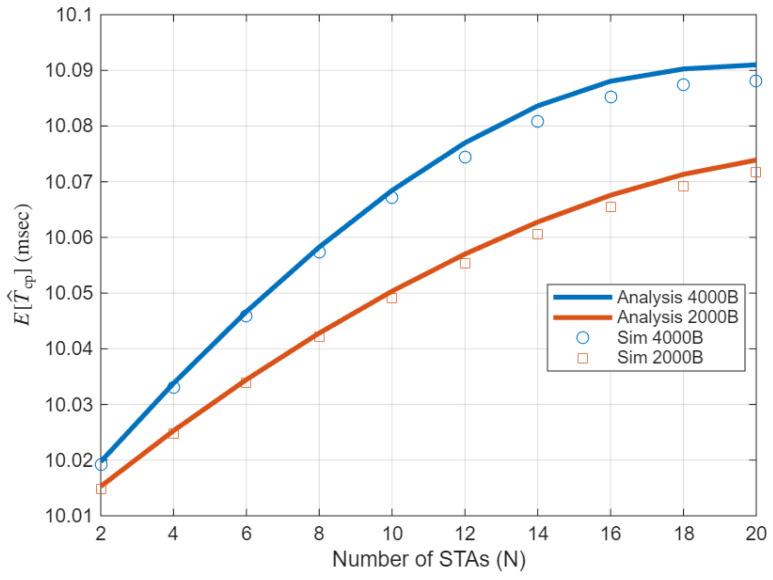
Validation of $E[{\hat{T}}_{\mathrm{cp}}]$: analysis vs. simulation across *N* and *L*.

Figure 8 cross-validates our analytical model for the effective contention period $E[{\hat{T}}_{\mathrm{cp}}]$. Both the analysis and the simulations use the physical/medium access control layer (PHY/MAC) and timing parameters in Table 1. Across N=2–20, the curves match closely and show a gentle increase of $E[{\hat{T}}_{\mathrm{cp}}]$ with *N*. The 4 kB case lies slightly above the 2 kB case, as longer transmissions increase the renewal residual and thus the effective contention period near the r-TWT SP boundary. These behaviors support our renewal (inspection paradox) treatment of boundary overrun and the EDCA Markov chain coupling adopted in this section.

### 3.4. Dimensioning the r-TWT Service Period Under Collisions and Packet Errors

To make the design reproducible and to avoid both under-provisioning (ST outage) and over-provisioning (bandwidth waste), practitioners need an explicit sizing rule for the service-period length $T_{r}$ that accounts for residual collisions and physical layer errors. This subsection closes that gap by deriving a constructive rule from the analytical elements established in the previous subsections.

Inside the ST service period, contention is removed by trigger-enabled access; nonetheless, residual failures can occur due to packet errors $p_{e}$.

The probability that the ST burst is delivered within *m* attempts is

(22) $$P_{\mathrm{succ}}\left(m\right)\;=\;1-{\left(p_{e}\right)}^{m},\;m\leq K_{\max}+1.$$

Given a target outage ${\hat{p}}_{o}$ per cycle, the minimum attempt budget is

(23) $$m^{★}\;=\;\left\lceil \frac{\ln({\hat{p}}_{o})}{\ln(p_{e})}\right\rceil ,$$

capped by $K_{\max}+1$. If $m^{★}>K_{\max}+1$, either the outage target or the PER must be relaxed, or the PHY rate/aggregation reduced.

Let *H* collect uplink triggering channel access overhead of the AP within an SP.

Choose the smallest integer $m^{★}$ satisfying (23) and set

(24) $$T_{r}\;\geq \;H\;+\;m^{★}\;T_{\mathrm{succ},st}\;+\;\Delta ,$$

where Δ≥0 is an optional safety margin (default Δ=0) for implementation jitter or timestamping granularity. This allocates just enough SP time to meet the outage target under the measured/assumed $p_{e})$ and the configured $K_{\max}$.

For a cycle of length $T_{\mathrm{st}}$ with guard band $T_{\mathrm{gb}}$ and service period $T_{r}$, the CP available to NST is

(25) $$T_{\mathrm{cp}}\;=\;T_{\mathrm{st}}\;-\;T_{\mathrm{gb}}\;-\;T_{r}.$$

Thus the ST–NST balance is tuned by jointly selecting $\left\{T_{\mathrm{gb}},T_{r}\right\}$. In managed deployments, bounding NST airtime near the boundary (e.g., TXOP/PPDU limits) allows $T_{\mathrm{gb}}$ to be sized to a high percentile of the bounded NST airtime distribution, minimizing the CP tax while preserving ST timeliness.

(i) When $p_{e}$ rises, either modestly increase $T_{r}$ via (24) or reduce ST aggregation (which lowers $T_{\mathrm{succ},st}$) to keep $P_{\mathrm{succ}}\left(m^{★}\right)$ unchanged. (ii) The AP can adapt $T_{r}$ over beacon intervals (or via action frames) using recent estimates of $p_{e}$, observed retries, and burst size *L*, thereby tracking the outage target without over-allocating the SP. (iv) Ensure $T_{r}\leq T_{\mathrm{st}}-T_{\mathrm{gb}}$ and that $m^{★}\leq K_{\max}+1$; otherwise, adjust the target or the PHY/aggregation.

## 4. Performance Evaluation

In this section, we discuss the performance evaluation of the proposed scheme. An event-driven MAC-level simulator, as described in [23,24], is used for the evaluation. The simulator was developed in MATLAB R2025a and implements IEEE 802.11 EDCA functions and relevant IEEE 802.11ax/be medium access control (MAC) layer features, such as TWT, QTP, and Triggered Uplink Access (TUA). The system parameters used for the simulation are listed in Table 1.

We evaluate the proposed scheme against EDCA under three configurations: Mode A = r-TWT only, Mode B = r-TWT + QTP, and Mode C = Mode B + Randomized Enqueue (RE). Unless otherwise noted, the stringent ST flow is trigger-enabled and served within an r-TWT SP in A/B/C, so ST determinism (throughput, outage, delay) is governed by PER and SP sizing and is essentially identical across these modes. We therefore place emphasis on the NST side: absolute NST throughput and EDCA-relative loss across the number of contenders (N={2,…,20}) and payload sizes ($L_{st},L_{nst}\in \left\{2,4\right\}$ kB). All simulation parameters and timing assumptions are summarized in Table 1.

**Figure 9:**
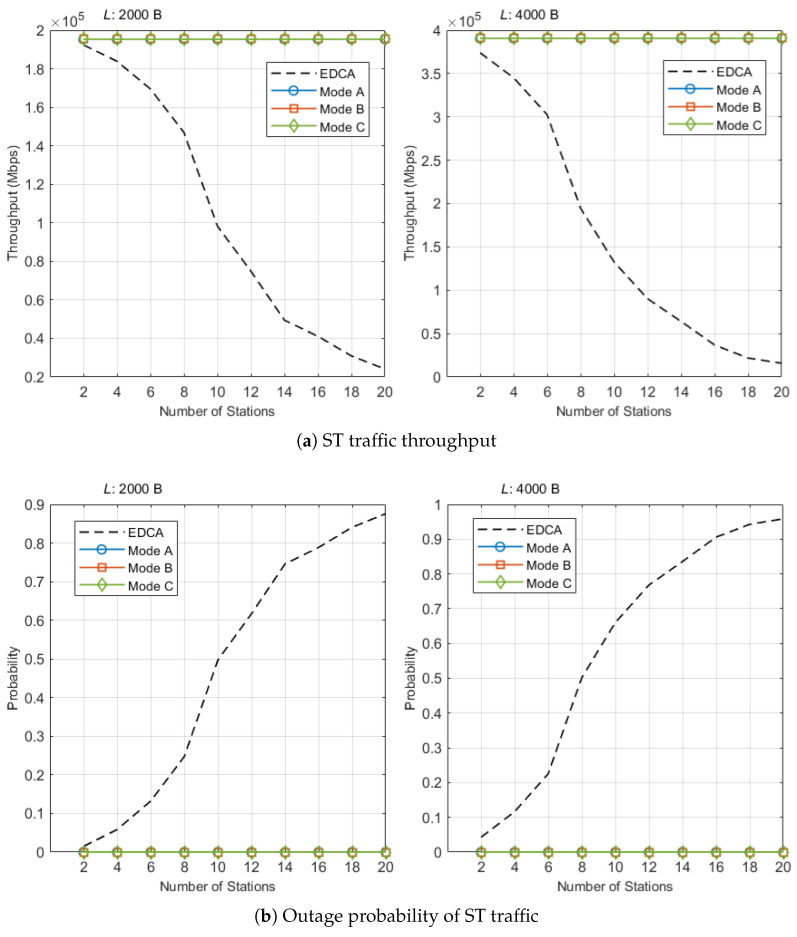
ST traffic performance evaluation.

We first evaluate the proposed scheme on Scheduled Traffic (ST) and compare it against conventional EDCA. Figure 9a and Figure 9b report ST throughput and outage probability, respectively. Because Modes A/B/C all serve ST within an r-TWT SP (trigger-enabled), their ST performance is expected to be identical to statistical noise, while QTP/RE only affect the NST side.

In Figure 9a, the theoretical ceiling $L_{st}/T_{st}$ is shown for reference. With $T_{st}=10$ TU (10.24 ms), the ceilings are 1.562 Mbps for $L_{st}=2$ kB and 3.125 Mbps for $L_{st}=4$ kB. Across all tested *N*, Modes A/B/C remain at this ceiling, confirming that r-TWT boundary protection guarantees on-time channel access for ST. By contrast, EDCA degrades sharply with contention. For $L_{st}=4$ kB, EDCA yields 1.557, 1.061, 0.722, 0.294, and 0.126 Mbps at N={8,10,12,16,20}, i.e., 49.8%, 34.0%, 23.1%, 9.4%, and 4.0% of the ceiling, respectively (falls below 25% by N=12). For $L_{st}=2$ kB, EDCA attains 1.176, 0.786, 0.599, 0.329, and 0.193 Mbps at the same *N* values, i.e., 75.3%, 50.4%, 38.3%, 21.1%, and 12.4% of the ceiling (25%-collapse by N=16). Representative points illustrate the gap: at N=10 and $L_{st}=4$ kB, Mode B achieves the ceiling 3.125 Mbps while EDCA achieves 1.061 Mbps (difference 2.064 Mbps); at N=16 and $L_{st}=2$ kB, Mode B remains at 1.562 Mbps while EDCA drops to 0.329 Mbps.

Figure 9b complements these results. Under EDCA, the outage probability (no success within the SP) rises with *N* and tends to 1 at high contention. In Modes A/B/C, we observed zero outages throughout the run. When no outages occur, we report the one-sided 95% upper bound by the rule of three:

(26) $${\hat{p}}_{o}\;\leq \;\frac{3}{\left\lfloor T_{\mathrm{sim}}/T_{st}\right\rfloor },$$

which evaluates to ${\hat{p}}_{o}\leq 7.68\times 10^{-4}$ for $T_{\mathrm{sim}}=40$ s (3906 cycles). Taken together, these results show that the proposed scheduling guarantees deterministic ST throughput and reliability across contention levels; differences among A/B/C appear only on the NST side and are analyzed in the next subsection.

**Figure 10:**
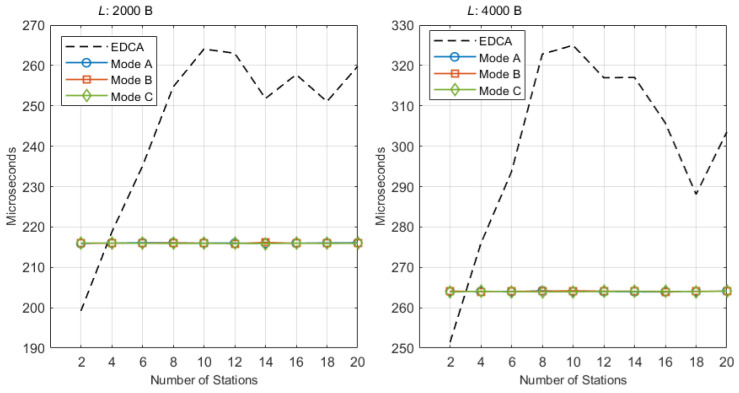
Average transmission delay of ST traffic.

Figure 10 reports the average transmission delay of ST traffic. Since Modes A/B/C all serve ST within an r-TWT service period with a trigger at the boundary, their ST delays are essentially identical and insensitive to the number of stations; QTP and Randomized Enqueue only influence NST behavior and do not alter the AP’s trigger-driven sequence inside the SP.

For $L_{st}=2$ kB, Modes A/B/C remain nearly flat at about 216 μs across the entire sweep (N=2…20). EDCA starts lower when the network is very lightly loaded and then increases with contention: from 199.18 to 259.79 μs. Thus EDCA is slightly smaller only at N=2 (about 17 μs below the proposed modes due to the absence of trigger overhead); from N≥4 the EDCA delay exceeds the proposed modes and the gap widens under contention. Over the practically relevant range N≥8, the average reduction achieved by Mode B relative to EDCA is about 39.4 μs.

For $L_{st}=4$ kB, Modes A/B/C form a similarly flat plateau around 264 μs. EDCA increases from 251.38 to 303.52 μs as *N* grows. Again, EDCA is slightly smaller only at N=2 (about 12–13 μs), but from N≥4, the proposed modes are equal or better; for N≥8, the average reduction of Mode B versus EDCA is about 50.7 μs, with representative gaps of 58.7 μs at N=8 and 60.8 μs at N=10.

Two remarks are important when interpreting EDCA. First, EDCA delays are computed over successfully delivered packets only; as contention rises, cycles that suffer an ST outage are excluded from the averaging, so the plotted EDCA curve does not reflect worst-case latency. Second, the flat delay plateaus of Modes A/B/C indicate that the guard band has been dimensioned at least as long as the maximum NST transmission on the link; had the guard been shorter than the longest NST airtime, an NST starting just before the guard could infringe the SP boundary and cause a visible bump near peak utilization. Under the present configuration, no such bump appears for $L_{st}\in \left\{2,4\right\}$ kB, and the proposed modes provide bounded and load-insensitive ST latency.

To complement Figure 10, Table 2 reports the variance of the ST transmission delay, defined as the sum of access delay and successful transmission time. To expose the impact of PHY errors, we include two error regimes: PER $=10^{-4}$ and $10^{-2}$. The EDCA baselines exhibit intrinsically high variance, and the variance grows with the number of contenders *N* as contention intensifies. In contrast, Modes A/B/C monopolize the channel during the SP and are therefore essentially insensitive to *N*; at PER $=10^{-4}$ they show very small variances with occasional small bumps from rare retransmissions. At PER $=10^{-2}$ the retry frequency increases and variances rise across all modes, although the scheduled modes remain orders of magnitude lower than EDCA. These results reinforce that the SP should be dimensioned with respect to the observed PER and payload size; as discussed in Section 3.4, using a conservative SP (or reducing ST aggregation) preserves the outage target without excessive bandwidth waste.

**Figure 11:**
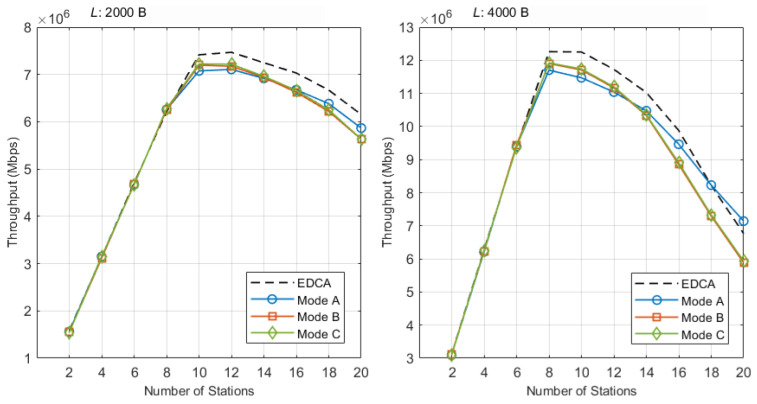
Absolute NST throughput vs. number of stations (*L* = 2/4 kB).

Figure 11 shows three operating regimes for NST throughput. (i) Low intensity (STA N=2–6): The medium is frequently idle and EDCA and Modes A/B/C deliver nearly the same NST capacity; the average gap is within about ±1% across payloads. (ii) System-peak throughput range (STA N=8–12): Where total system throughput is maximized, Mode C (r-TWT + QTP + RE) consistently minimizes NST loss by smoothing the CP onset after each SP. Averaged over N={8,10,12}, the NST reduction relative to EDCA is about −1.6% at $L_{st}=2$ kB and −3.8% at $L_{st}=4$ kB, smaller than Mode A (−2.9%, −5.6%) and Mode B (−2.1%, −4.0%). The detailed values in Table 3 confirm this. For $L_{st}=2$ kB, at N=8, the three modes are essentially indistinguishable from EDCA (EDCA 49.66 Mbps, Mode A 50.06 Mbps, Mode B 49.99 Mbps, Mode C 50.23 Mbps), while at N=10,12, Mode C shows the smallest losses (−2.6% and −3.4%). For $L_{st}=4$ kB, Mode C again yields the smallest losses at N=8,10,12 (−2.8%, −4.2%, −4.5%, respectively), improving on Mode A (−4.5%, −6.4%, −5.8%) and Mode B (−2.9%, −4.4%, −4.8%). (iii) High intensity (STA N=14–20): NST throughput drops for all schemes as collisions and reservation overhead rise; here, Mode A (r-TWT only) often shows the smallest EDCA-relative loss because it does not pay the quiet-time overhead. Consistent with this, the winner table (Table 4) selects Mode C in the system-peak range for both payloads, while favoring Mode A in the highest-load band.

Additional observations from the absolute NST throughput plot in Figure 11 are as follows. Up to about 10 STAs, all three modes track EDCA closely, indicating that the capacity cost of determinism is small at low–mid density. Representative points: At N=10 and $L_{st}=4$ kB, EDCA is 98.00 Mbps while Modes A, B, and C attain 91.78, 93.67, 93.90 and Mbps; at N=8 and $L_{st}=2$ kB, EDCA is 49.66 Mbps and the modes are 50.06, 49.99, and 50.23 Mbps, essentially indistinguishable. Beyond 12 STAs, all curves decline as contention rises; the tables make clear where Mode C brings the most benefit (system-peak range, via desynchronization and burst smoothing) and where Mode A becomes preferable (extreme load, where avoiding quiet-time overhead helps preserve NST airtime).

Table 3a,b summarize the system-peak range at $L_{st}\in \left\{2,4\right\}$ kB. For $L_{st}=2$ kB, the EDCA baseline is 49.66/59.33/59.75 Mbps at N=8/10/12, and Mode C yields 50.23/57.77/57.74 Mbps with relative deltas of about +1.1%, −2.6%, and −3.4%; these are the smallest reductions among A/B/C at N=10,12. For $L_{st}=4$ kB, EDCA is 98.08/98.00/93.81 Mbps, and Mode C gives 95.37/93.90/89.60 Mbps, corresponding to −2.8%, −4.2%, and −4.5%, again the smallest loss in each column. The winner table (Table 4) aggregates by STA band and payload: at low intensity, all modes are effectively tied; in the system-peak range, Mode C minimizes loss at both payloads; at high intensity, Mode A becomes the least costly due to the absence of quiet-time overhead. This pattern matches the design intent: QTP desynchronizes contenders and RE removes burstiness where it matters most, while avoiding QTP when the contention-driven overhead would dominate.

**Figure 12:**
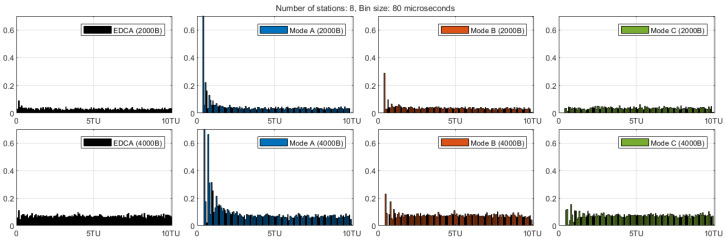
Collision density at CP onset (*N* = 8, bin width 80 μs).

The previous results established that r-TWT guarantees on-time ST transmission and that Mode C preserves NST capacity best in the system-peak range (N=8–12). To explain why these macroscopic trends emerge, Figure 12 examines the post-SP micro-dynamics. It partitions the CP timeline (origin at the ST acknowledgment (ACK)) into 80-μs bins and reports, for each bin, the average number of NST collisions per cycle. Let $C_{b}$ denote collisions/cycle in bin *b*. Define the CP-start bin index as

(27) $$b_{s}\;≜\;\min\left\{\;b\;|\;C_{b}>0\;\right\},$$

i.e., the first active (non-zero) bin where the CP actually begins (typically $b_{s}\;=\;0$, but it can be >0 under SP overrun). We write $C_{b}$ for the collisions/cycle in bin *b*, define the boundary-bin value $C_{\mathrm{start}}\;≜\;C_{b_{s}}$, and measure the early-window collision burden from the true CP start as

(28) $$A_{b_{s}:K}^{\mathrm{col}}\;≜\;\sum_{b=b_{s}}^{b_{s}+K}C_{b},$$

which aggregates the collisions/cycle over the first (K+1) bins anchored at $b_{s}$ (e.g., K=5,20 correspond to 0.48 ms and 1.68 ms). Quantitative values for these metrics at N=8 and $L_{st}\in \left\{2,4\right\}\;\mathrm{kB}$ are summarized in Table 5, which complements the histogram in Figure 12. Large $C_{\mathrm{start}}$ and large $A_{b_{s}:K}^{\mathrm{col}}$ reveal the transient collision pressure right after the SP, which directly reduces NST throughput at CP onset. We use N=8 (system throughput peak) where mode differences are most visible.

EDCA does not separate SP and CP, so NST contention runs continuously around the scheduled ST instant. Consequently the collision profile shows a moderately elevated but temporally spread density near the anchor, rather than a sharp boundary spike. This continuous contention explains the rapid growth of EDCA’s ST delay with the number of contenders and serves as a baseline for quantifying collision suppression in the proposed modes.

In Mode A, non-participating STAs keep counting down during the SP; many reach small residual counters and then attempt almost simultaneously when the CP actually starts. Accordingly, the first active bin already carries nearly one collision per cycle: $C_{\mathrm{start}}\;=\;1.06$ at $L_{st}\;=\;2$ kB and $C_{\mathrm{start}}\;=\;0.97$ at $L_{st}\;=\;4$ kB. Early-window burdens anchored at $b_{s}$ are large—approximately $A_{b_{s}:5}^{\mathrm{col}}\;\approx \;2.40$ and 2.10, and $A_{b_{s}:20}^{\mathrm{col}}\;\approx \;4.00$ and 3.70 for 2 kB and 4 kB, respectively. This near-certain boundary collision explains the NST capacity dip of Mode A in the peak range.

Mode B (r–TWT + QTP) desynchronizes residual backoff counters during the SP, but NST frames that arrive during the SP are released at once when QTP ends, yielding a strong yet time-spread burst of contention at CP start. QTP halts backoff drain for non-participating stations during the SP, so residual counters do not collapse to near-zero; meanwhile, NST arrivals accumulate in queues. When QTP ends, these non-idle stations resume together, producing a strong but time-spread burst across the first few CP bins rather than a single boundary spike. This de-synchronizes counters yet still releases buffered demand at CP start, which is why Mode B improves stability over Mode A but does not suppress the early-window burden as effectively as Mode C Approximate reads (collisions/cycle), anchored at $b_{s}$: at $L_{st}=2$ kB, $C_{\mathrm{start}}\;\approx \;0.80$, $A_{b_{s}:5}^{\mathrm{col}}\;\approx \;2.20$, $A_{b_{s}:20}^{\mathrm{col}}\;\approx \;3.90$; at $L_{st}=4$ kB, $C_{\mathrm{start}}\;\approx \;0.90$, $A_{b_{s}:5}^{\mathrm{col}}\;\approx \;2.40$, $A_{b_{s}:20}^{\mathrm{col}}\;\approx \;\;4.10$.

Mode C (r–TWT + QTP + Randomized Enqueue) disperses that boundary burst for short ST airtime by spreading buffered arrivals over the CP, but the benefit weakens under SP overrun because arrivals during the overrun are not shaped and re-create an early spike. Randomized Enqueue (RE) spreads buffered NST arrivals over the CP to soften Mode B’s boundary micro-burst. With $L_{st}\;=\;2$ kB (short ST airtime, negligible overrun), the boundary pressure drops: $C_{\mathrm{start}}\;\approx \;0.55$, $A_{b_{s}:5}^{\mathrm{col}}\;\approx \;1.80$, $A_{b_{s}:20}^{\mathrm{col}}\;\approx \;3.60$. With $L_{st}=4$ kB, longer ST airtime raises the incidence of r-TWT SP overrun. Because QTP ends at the scheduled SP boundary, NST arrivals during the overrun interval are not shaped by RE and remain as regular EDCA arrivals; when the ST ACK finally releases the medium, they contend promptly, partially restoring the boundary spike: $C_{\mathrm{start}}\;\approx \;0.78$, $A_{b_{s}:5}^{\mathrm{col}}\;\approx \;2.20$, $A_{b_{s}:20}^{\mathrm{col}}\;\approx \;4.00$. This explains why Mode C’s NST advantage shrinks at larger payloads: overrun short-circuits RE right at the boundary.

## 5. Discussion

### 5.1. Overview and Alignment with Objectives

The evaluation results indicate that our objectives have been met. Despite operating over a contention-based IEEE 802.11 WLAN, the composition of Restricted Target Wake Time (r-TWT) and Quiet Time Period (QTP) establishes a functional guard band that reliably protects the Scheduled Traffic (ST) service period (SP) while avoiding synchronized collision after SP ends. Furthermore, by leveraging trigger-enabled TWT, the access point (AP) can explicitly solicit the designated ST frames from stations (STAs) within the SP, thereby realizing the semantics of Time-Aware Shaper (TAS) gate control on top of WLAN. Importantly, only the contention period (CP) boundary dynamics are reshaped; the steady-state CP remains opportunistic for Non-Scheduled Traffic (NST), preserving overall WLAN efficiency. An optional Randomized Enqueue (RE) policy can further smooth early-CP micro-bursts without altering the PHY.

### 5.2. Interfacing with the TSN Control Plane

**Figure 13:**
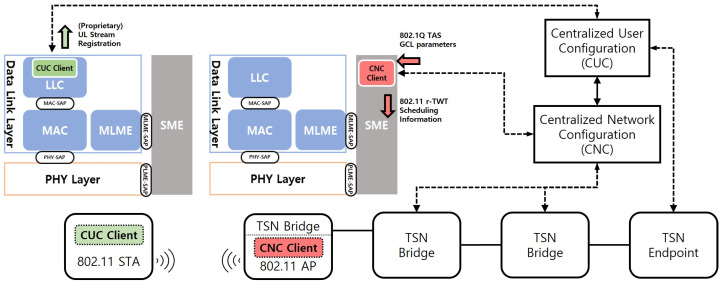
Proposed reference model for TSN-WLAN interfacing.

For the proposed scheme to operate within a real-world TSN environment, a standardized interfacing framework between the TSN control plane and the WLAN (AP/STA) is required. As no such standard currently exists for IEEE 802.11, this paper proposes a reference model based on standard architectures, as shown in Figure 13.

This model logically connects the centralized configuration model of TSN with the management entities of IEEE 802.11. The primary components and their operational flow are as follows:

1. CUC Client @ STA: An application generating ST traffic interfaces with a CUC (Centralized User Configuration) Client located at the Logical Link Control (LLC) layer of the station (STA). When a new ST stream is created, the CUC Client gathers its requirements—such as period, latency, and data size—and sends a Stream Registration Request to the central CUC server.
2. CNC Client @ AP: Once the CUC registers the stream, it instructs the CNC (Centralized Network Configuration), which manages the overall network schedule, to configure the necessary paths and schedules. To schedule the WLAN segment, the CNC communicates with a CNC Client residing within the Station Management Entity (SME) of the access point (AP).
3. Parameter Translation: The core function of the CNC Client is to translate the TAS parameters received from the CNC into equivalent TWT parameters suitable for the wireless environment. This translation process acts as the essential bridge between the two heterogeneous network technologies. In IEEE 802.1Qbv, each gate control entry (GCE) specifies a time interval and gate state; the mapping rules are summarized in Table 6.
4. Schedule Configuration: The TWT parameters, now translated by the CNC Client, are passed to the AP’s MLME (MAC Sublayer Management Entity). The MLME then uses these parameters to configure the final r-TWT schedule and disseminates it through Beacon frames or other management frames.

Through this model, the proposed scheme provides a concrete and standards-compliant method for interfacing with a standard TSN control plane, enabling the extension of end-to-end deterministic communication into the wireless domain.

### 5.3. Implementation Considerations

Building on this control-plane mapping, we outline a pragmatic realization path that preserves standard compliance and avoids chipset modifications. In practice, the AP and STAs share a common time base via IEEE 802.11 TSF bridged to a generalized precision time protocol (gPTP, IEEE 802.1AS), and this alignment lets the AP span QTP over the entire guard band $T_{\mathrm{gb}}$ immediately before each SP and then issue trigger-enabled access at the SP start, thereby enforcing TAS-like gating semantics on WLAN.

On the implementation side, most Wi-Fi 6/6E/7 MAC functions reside in chipset firmware or ASIC, which makes deep MAC changes difficult for third parties. We therefore adopt a split design that requires no PHY changes:

- AP side: The SME–MLME pipeline exposes r-TWT/QTP schedule attributes (for example, via MLME-SAP), allowing firmware to advertise SP/GB timing in Beacons/Action frames and to issue trigger frames during the SP for the intended AC/TID.
- STA side (RE): While RE for NST is not yet standardized, SP/GB timing is visible to the host via SAP attributes, so a proprietary driver-level policy can pace NST dequeues around the CP restart. When the MLME indicates an imminent SP end (or CP start), the driver perturbs dequeue times (through LLC–MAC SAP interaction) to spread early-CP transmissions over a short window, implementing RE without modifying chipset firmware.

Putting the pieces together, the control flow is (i) CNC/CUC → AP/SME: deliver the TAS Gate Control List (GCL); (ii) the SME maps the GCL to {r-TWT $T_{\mathrm{sp}}$, QTP $T_{\mathrm{gb}}$, trigger pattern}; (iii) the AP/MLME advertises the schedule (Beacon/Action), enrolls ST STAs (TWT setup), and executes trigger-enabled ST during the SP; and (iv) the STA/MLME consumes the schedule attributes and, if acting as NST, applies RE pacing at CP restart via LLC–MAC SAP.

Finally, from a standardization perspective, IEEE 802.11 Working Group activities on TSN interworking and next-generation WLAN are actively discussing tighter time-aware operations; while RE can be prototyped as above, the most robust path is to standardize this functional suite—r-TWT scheduling profiles, QTP alignment semantics over the guard band, and an optional RE/pacing primitive (or MLME hint)—so that APs and STAs interoperate without proprietary hooks.

### 5.4. Limitations and Threats to Validity

First, there is an inherent trade-off between the timeliness of ST deliveries and the throughput tax introduced by the functional guard band (GB). Let $T_{\mathrm{cycle}}$ denote the ST cycle period. The capacity loss scales with the GB fraction,

(29) $$\Delta \;\rho \approx \rho_{\mathrm{CP}}\cdot \frac{T_{\mathrm{gb}}}{T_{\mathrm{cycle}}},$$

where $\rho_{\mathrm{CP}}$ is the attainable utilization during CP-absent GB. As the variance of NST PPDU airtimes increases, a GB sized to cover the upper tail of NST transmissions must grow, further reducing efficiency; this sensitivity is most visible when large, highly aggregated NST frames are permitted near the SP boundary. In managed industrial or enterprise environments, however, GB efficiency can be restored through administrative controls that bound NST airtime: (i) enforce a per-BSS TXOP limit size, (ii) constrain application chunk sizes and pacing for traffic traversing the ST BSS, and (iii) prefer MCS/RU policies that limit worst-case airtime near the SP boundary. With these controls in place, $T_{\mathrm{gb}}$ can be dimensioned conservatively (for example, to a high percentile of the bounded NST airtime distribution) without incurring an undue throughput penalty, preserving the ST timeliness–throughput balance.

A second limitation concerns dense overlapping BSS (OBSS) environments: external STAs may not honor the ST BSS’s QTP or r-TWT membership, so SP protection can be partially undermined by co-channel contenders. A systematic, quantitative treatment of OBSS impact—spanning density, channel reuse, BSS coloring/spatial reuse, and compliance rates—is left to future work. In managed industrial/enterprise deployments, much of the risk can be mitigated by (i) channel planning and spatial placement to avoid overlapping operating channels across co-located BSSs, (ii) stricter device policy within the ST BSS (for example, requiring QTP honor and r-TWT membership), and (iii) conservative perimeter settings (power, CCA) near SP boundaries. In addition, coordinated r-TWT across BSSs—currently under discussion for IEEE 802.11bn—aims to provide cross-BSS SP protection and can further reduce boundary collisions when available.

Beyond cross-BSS effects, residual variability can arise under elevated PER, time synchronization errors that misalign QTP and SP boundaries, or in-BSS legacy stations that ignore QTP. Our evaluation is simulation-based; heterogeneous over-the-air testbeds are left for future validation. Extremely large payloads may force conservative choices for $T_{\mathrm{gb}}$ and $T_{\mathrm{sp}}$ unless administratively bounded as discussed above.

### 5.5. Cross-Technology Comparison and Implications for Our Design

Consistent with our control-plane mapping in Section 5.2, we position the proposed WLAN path against contemporary 5G/URLLC deployments using quantitative evidence from industrial studies. Under the 3GPP indoor factory framework, a head-to-head evaluation finds that Wi-Fi 6 satisfies sub-1 ms application targets only at very low offered load and in interference-free spectra, whereas licensed 5G NR generally delivers the strongest sub-1 ms performance together with roughly 2–4× higher spectral efficiency; as latency requirements relax to 10–100 ms, the performance gap narrows and Wi-Fi becomes practical for local domains [25]. Complementing this, an indoor factory testbed reports that Wi-Fi 6 achieves the lowest median latency but exhibits heavy-tailed delay and higher loss—especially under mobility where the 99.99th-percentile latency can exceed ∼120 ms—while 5G SA yields stable, bounded tails. Furthermore, 5G+Wi-Fi multi-connectivity collapses the tails toward the 5G distribution, and the testbed itself relies on a private 5G deployment with an in-house 5G SA core, underscoring the core-integrated, centrally scheduled nature of that path [26].

In contrast, our path is purpose-built for owner-operated, local TSN islands—room- or cell-scale industrial or enterprise domains where the set of STAs is known and managed, the layout is fixed, and time sync (TSF bridged to IEEE 802.1AS) is available at the AP. Typical targets include factory cells and lines, labs and test benches, clean rooms, and campus rooms where TSN micro-windows (on the order of a few to a few tens of milliseconds) are needed but deploying licensed spectrum, a cellular RAN, or a 5G core is neither necessary nor cost-effective. In these environments, administrators can apply standard WLAN hygiene—channel planning and spatial separation to avoid OBSS overlap, policy control to bound PPDU size or TXOP near SP boundaries, and monitoring of PER to adapt $T_{\mathrm{sp}}$—so that deterministic windows are delivered at low CAPEX/OPEX on commodity Wi-Fi 6/7 gear. The approach leverages high per-link bandwidth (wide channels and, where available, 11be features such as multi-link operation) for bursty ST payloads, requires no PHY changes, and integrates with the TSN control plane at the AP management layer (CUC/CNC → TAS GCL → TWT/QTP/trigger mapping). ST STAs participate as TWT members with trigger-enabled uplink; NST STAs can remain unchanged, with an optional driver-level RE policy for variance reduction. The intended scope is indoor, low-mobility cells with strong administrative control; wide-area mobility and carrier-grade SLAs are better served by centrally scheduled licensed systems, making the two ecosystems complementary rather than competing.

An industrial review similarly frames 5G as a core-integrated platform for wide-area, SLA-driven scenarios and Wi-Fi 7 as a cost-effective enabler for local deterministic windows via TWT or r-TWT and EHT features (e.g., wide channels and multi-link operation), which aligns with our target environment and the functional guard band approach used here [4].

## 6. Conclusions

This paper presented a novel transmission scheme that orchestrates r-TWT, QTP, and Randomized Enqueue policy to deliver deterministic Scheduled Traffic (ST) over WLAN while limiting the impact on Non-Scheduled Traffic (NST). The roles are separated by design: r-TWT prevents CP overrun (functional guard band), QTP removes synchronized backoff collapse during the SP, and RE spreads buffered NST arrivals across the CP to soften the boundary micro-burst. A control-plane mapping from TAS to TWT/QTP was also outlined.

Our analysis used renewal theory for CP overrun and yielded closed forms for SP underrun/overrun under a geometric error model, with or without early termination. On the NST side, a standard EDCA Markov chain was aligned to the r-TWT/QTP timeline and augmented with RE via an effective arrival-shaping abstraction.

Evaluation confirms the intended behavior: Modes A/B/C keep ST throughput at the ceiling $L_{st}/T_{st}$ with zero observed outage and bounded delay. For NST, the results are regime-dependent in a way that guides deployment choices. In the practically relevant operating region, where the system throughput peak around 8–12 STAs, Mode C (r-TWT + QTP + RE) consistently minimizes EDCA-relative loss (about −1.6% at 2 kB and −3.8% at 4 kB on average), thus preserving NST capacity while guaranteeing deterministic ST. Under extreme loads (14–20 STAs), Mode A (r-TWT only) may appear preferable thanks to lower reservation overhead, but that regime already departs from capacity-optimal operation and is less representative of intended TSN use. Focusing on the practically relevant system-peak range (about 8–12 stations), we recommend Mode C (r-TWT + QTP + RE) as the default: it preserves NST capacity while guaranteeing deterministic ST. Under sustained extreme loads (14–20 stations), where the network already departs from the capacity-optimal regime, the lighter r-TWT-only policy (Mode A) can be used as a fallback when NST capacity must be prioritized.

Future work includes (i) adaptive guard/QTP sizing driven by live measurements and PER estimates, (ii) fairness across multiple NST ACs and flows under mixed payloads, and (iii) impact analysis under overlapping BSS (OBSS), including how neighboring BSS activity interacts with r-TWT/QTP timing and RE shaping across contention domains.

## Figures and Tables

**Table 1:** Simulation parameters.

Parameter	Value
Evaluated modes	EDCA, Mode A (r-TWT), Mode B (r-TWT + QTP),
	Mode C (r-TWT + QTP + RE)
Number of STAs	2, 4, 6, 8, 10, 12, 14, 16, 18, 20
Transmission bandwidth	20 MHz
OFDM symbol duration (11be)	16 μs
Preamble duration (Legacy)	40 μs
Preamble duration (11be SU/TB)	48 μs
Modulation and coding scheme (MCS) index (Control/data frame)	4/2
Trigger frame MAC payload	38 Bytes
Payload size ($L_{nst}$ = $L_{st}$ = *L*)	2000/4000 Bytes
ST transmission sequence	[Legacy]Trigger Frame ++ [11be trigger-based (TB) PPDU]Data ++ [11be SU]ACK
NST transmission sequence	[11be single user (SU) PPDU]Data ++ [11be SU]ACK
Packet Error Rate ($p_{e}$)	$10^{-4}$
Slot Time	9 μs
Short inter-frame spacing (SIFS)	16 μs
AIFS number (AIFSN) (for AC_VO, VI, BE, BK)	[2, 2, 3, 7]
$CW_{min}$ (for AC_VO, VI, BE, BK)	[7, 15, 31, 31]
$CW_{max}$ (for AC_VO, VI, BE, BK)	[15, 31, 1023, 1023]
Retry Limit (for AC_VO, VI, BE, BK)	[7, 7, 7, 7]
ST service interval ($T_{st}$)	10,240 μs (10 time unit (TU))
r-TWT SP duration ($T_{r}$)	0.25 TU
Guard band duration	Maximum NST transmission sequence duration
RE policy	Uniform randomization over the next CP interval
Simulation duration	40,000 ms

**Table 2:** Variance of ST transmission delay (access delay + successful transmission time) under varying payloads and packet error rates.

Payload	PER	EDCA *N* = 2	EDCA *N* = 10	EDCA *N* = 20	Mode A	Mode B	Mode C
2000 B	$10^{-4}$	2740.0	6370.0	6330.0	6.6416	10.53903	10.60567
2000 B	$10^{-2}$	3230.0	5980.0	6180.0	400.18468	350.14171	374.30620
4000 B	$10^{-4}$	3570.0	6910.0	7210.0	12.73763	9.85574	12.68245
4000 B	$10^{-2}$	3660.0	6890.0	4970.0	532.89593	576.43240	600.65931

**Table 3:** System-peak NST throughput at N∈{8,10,12} for L∈{2,4} kB.

(a) ***L*** = 2 kB							
*N*	EDCA (Mbps)	A (Mbps)	ΔA[%]	B (Mbps)	ΔB[%]	C (Mbps)	ΔC[%]
8	49.66	50.06	0.8	49.99	0.7	50.23	1.1
10	59.33	56.60	−4.6	57.57	−3.0	57.77	−2.6
12	59.75	56.86	−4.8	57.39	−3.9	57.74	−3.4
**(b)** ***L* = 4 kB**							
*N*	EDCA (Mbps)	A (Mbps)	ΔA[%]	B (Mbps)	ΔB[%]	C (Mbps)	ΔC[%]
8	98.08	93.61	−4.5	95.20	−2.9	95.37	−2.8
10	98.00	91.78	−6.4	93.67	−4.4	93.90	−4.2
12	93.81	88.33	−5.8	89.34	−4.8	89.60	−4.5

**Table 4:** Mode with least average NST reduction per STA band and *L*.

*L* (B)	STA Band	Best Mode	Δ[%] (Avg)
2 K	Low (N = 2–6)	A	−0.6
2 K	System-peak (N = 8–12)	C	−1.6
2 K	High (N = 14–20)	A	−4.6
4 K	Low (N = 2–6)	B	0.0
4 K	System-peak (N = 8–12)	C	−3.8
4 K	High (N = 14–20)	A	−0.9

**Table 5:** Collision density near the CP boundary (*N* = 8, bin width Δ = 80 μs).

(a) Payload $L_{\mathrm{st}}\;=\;2$ kB					
Scheme	$C_{\mathrm{start}}$	$A_{b_{s}:2}$	$A_{b_{s}:5}$	$A_{b_{s}:10}$	$A_{b_{s}:20}$
Mode A (r-TWT)	1.06	1.70	2.40	3.10	4.00
Mode B (r-TWT + QTP)	0.80	1.30	2.20	3.00	3.90
Mode C (r-TWT + QTP + RE)	0.55	0.95	1.80	2.70	3.60
**(b) Payload** $L_{\mathrm{st}}\;=\;4$ **kB**					
Scheme	$C_{\mathrm{start}}$	$A_{b_{s}:2}$	$A_{b_{s}:5}$	$A_{b_{s}:10}$	$A_{b_{s}:20}$
Mode A (r-TWT)	0.97	1.50	2.10	2.80	3.70
Mode B (r-TWT + QTP)	0.90	1.50	2.40	3.20	4.10
Mode C (r-TWT + QTP + RE)	0.78	1.25	2.20	3.10	4.00

**Table 6:** Mapping of TAS parameters to WLAN parameters and dimensioning rules.

TAS Parameter (from CNC)	WLAN Parameter (AP MLME)	Description
BaseTime	Target Wake Time (TWT)	Absolute schedule anchor; start of the first r-TWT SP.
CycleTime	Wake Interval	Period; equals the ST service interval.
GCE TimeInterval (ST slot)	Wake Duration (r-TWT)	ST slot length → r-TWT SP duration.
GCE GateState (ST queue open)	TWT Member & Trigger-enabled	Single STA as r-TWT member; AP triggers intended AC/TID at SP start.
Guard Band (length)	Restricted TWT & QTP alignment (boundary protection)	GB is dimensioned by the longest NST transmission time on the link so that no NST TXOP can spill into the SP. QTP starts one such NST transmission time before each SP and remains active until the end of SP.

## Data Availability

The original data presented in this study are openly available at https://doi.org/10.22711/idr/1106.
